# Influence of Anxiety/Depression on the Subjective Evaluation of Cough in Patients with Chronic Obstructive Pulmonary Disease and Obesity

**DOI:** 10.3390/medicina55050134

**Published:** 2019-05-14

**Authors:** Evgeniy S. Ovsyannikov, Sergey N. Avdeev, Andrey V. Budnevsky, Yanina S. Shkatova

**Affiliations:** 1 Department of Faculty Therapy, Voronezh State Medical University, 394036 Voronezh, Russia; ovses@yandex.ru (E.S.O.); budnev@list.ru (A.V.B.); 2 Pulmonology Department, I.M. Sechenov First Moscow State Medical University, 119992 Moscow, Russia; serg_avdeev@list.ru

**Keywords:** chronic obstructive pulmonary disease, obesity, depression, anxiety, cough monitoring

## Abstract

*Background and objectives:* Obesity and anxiety and/or depression are common comorbidities in patients with chronic obstructive pulmonary disease (COPD). For doctors treating COPD, cough has a certain importance as a symptom. The purpose of this study was to figure out how obesity and anxiety/depression may influence the subjective assessment of cough. *Materials and Methods:* 110 patients with COPD participated in the study. The patients were divided into two groups, one including obese patients, and the other including patients with normal body weight. All patients filled out the hospital anxiety and depression scale (HADS) questionnaire, evaluated the severity of their cough by using visual analogue scale (VAS) on the 1st and 10th day of treatment, and underwent a 12 h cough monitoring with a special cough monitoring device both on the 1st and the 10th day of treatment. *Results:* The severity of anxiety according to the HADS in patients with COPD and normal body weight was significantly higher than in patients with COPD and obesity, corresponding to 9.25 ± 1.37 and 8.20 ± 1.18 points, respectively (*p* = 0.0063). The patients with normal body weight and obesity, but without anxiety and depression, subjectively noted an improvement in their well-being on the 10th day of treatment (*p* = 0.0022, *p* = 0.0021, respectively). In subgroups with normal body weight and obesity with anxiety and/or depression, the mean values for VAS on day 10 did not change significantly (*p* = 0.1917, *p* = 0.1921, respectively). Also, patients from the subgroup with normal body weight and anxiety/depression had a significantly higher assessment of their cough on day 10 than obese patients with anxiety/depression (*p* = 0.0411). The VAS values correlated positively with the actual amount of cough (*r* = 0.42, *p* = 0.0122 and *r* = 0.44, *p* = 0.0054, respectively) in patients without anxiety and/or depression, while in patients with anxiety and/or depression, there was an inverse correlation between VAS values and cough (*r* = −0.38, *p* = 0.0034 and *r* = −0.40, *p* = 0.0231). *Conclusions:* It is important to diagnose and treat anxiety and depression in patients with COPD for a better prognosis and higher efficacy of medical treatments. While treating such patients, it is preferable to use a cough monitoring device for objective assessments, since the patients may exaggerate or underestimate their symptoms.

## 1. Introduction

Chronic obstructive pulmonary disease (COPD) is one of the leading causes of chronic morbidity and mortality worldwide [1,2]. In recent years, special attention has been paid to comorbidities in COPD, since it has been proven that the presence of comorbidities contributes to a more severe course of COPD and an increase of morbidity and mortality in such patients [3,4]. Comorbid diseases can occur in patients with any degree of airflow restriction, not only in patients with severe COPD [5]. The prevalence of comorbidities is quite high: more than 50% of patients with COPD have 1–2 comorbidities, 15.8% have 3–4, and 6.8% have more than 5 [6]. In general, in patients with COPD, attention is paid to the following pathologies: cardiovascular diseases, osteoporosis, skeletal muscle dysfunction, weight loss [7,8,9]. However, it is important to take into account such conditions as anxiety and depression, which, according to several studies, significantly affect the course of the disease. When comparing the prevalence of anxiety and depression in the population and among patients with COPD, it was found that, on average, depression occurs in 10% of the population in the majority of countries, while 40% of patients with COPD suffer from depression and/or increased anxiety [10]. According to a few studies, depression is often combined with anxiety; moreover, the risk of anxiety disorder in patients with COPD and depression is seven times higher than the risk in patients with COPD who do not suffer from depression [11]. According to a study by Lacasse Y. et al. in outpatients with COPD, depression ranges from 7% to 80%, and anxiety from 2% to 80% [12]. The prevalence of generalized anxiety disorder ranges from 10% to 33% and that of panic attacks from 8% to 67%. In stable COPD patients, the prevalence of clinical depression ranges from 10% to 42%, and that of anxiety from 10% to 19% [13]. There is a possibility that patients with anxiety disorders and depression may inadequately assess their somatic complaints (exaggerating or minimizing the significance of the symptoms), which in turn may lead to a decrease in treatment effectiveness and a worse prognosis of somatic diseases [14]. All of the above happens because of a cognitive dysfunction. An improvement in cognitive performance in depressed subjects may be achieved through repetitive transcranial magnetic stimulation (rTMS), aside from standard pharmacological treatments [15].

Among all comorbidities in COPD, obesity is also very important, especially since its prevalence continues to increase. A few studies have reported a higher prevalence of obesity in people with COPD compared with patients without it. The number of patients with COPD and obesity was 25% in Canada [16], 23% in Latin America [17], 18% in Holland [18], and 54% in a small sample of patients in California, USA [19]. Data on the obesity effect on the COPD course are contradictory, but the prognosis of patients with COPD and obesity is more favorable (“obesity paradox”) [20]. At the same time, there are insufficient data on the relationship between obesity and anxiety, although many studies have noted an association between obesity and panic disorder, mainly in women [21]. In the meanwhile, depression is stated to be simultaneously a predictor and a consequence of obesity in the population as a whole [22]. According to a meta-analysis by Luppino F.S. et al., where authors examined available studies on the association between obesity and depression, obese patients had a 55% increased risk of developing depression over time, while patients suffering from depression had a 58% increased risk of becoming obese [23].

Cough is one of the most important symptoms in patients with COPD, and at this point, the diagnosis of cough and its causes is performed with some difficulties, since visual analogue scales (VAS) and specially designed questionnaires (for example, the Leicester Cough Questionnaire) are very subjective [24]. Therefore, there is a need for objective ways to evaluate cough.

Considering all of the above, the purpose of this study was to evaluate how objective patients with COPD and obesity can be when assessing the severity of their cough by using a cough monitoring device.

## 2. Materials and Methods

The study included patients with COPD: 70 men (63.64%) and 40 women (36.36%). According to the Global Initiative for Chronic Obstructive Lung Disease (GOLD) classification, all patients belonged to the group D, GOLD 2–4. COPD diagnosis was made according to GOLD (2018); complaints, anamnesis data, objective status data, spirometry (with a 400 mg salbutamol test) were analyzed. We had the following inclusion criteria: COPD exacerbation at the time of the study, informed patient consent for voluntary participation in the study. Exclusion criteria from the study were patient participation in any other study, cancer, pneumonia, tuberculosis, sarcoidosis, bronchial asthma, interstitial pulmonary fibrosis, acute coronary syndrome, chronic heart failure, chronic kidney disease, exacerbations of other chronic diseases.

All patients gave their informed consent for inclusion before they participated in the study. The study was conducted in accordance with the Declaration of Helsinki, and the protocol was approved by the Ethics Committee of Voronezh State Medical University (protocol no. 1 from 21 February 2018).

All patients were admitted to the hospital and received standard medical treatment according to the GOLD (2018), precisely: short-acting β2-agonists (with increased dosage), long-acting β2-agonists (with the use of a nebulizer for drug delivery to the respiratory tract), antibiotic therapy (III generation cephalosporins, in case of intolerance to macrolides).

In addition to standard examination methods, on the day of admission, all patients filled out the hospital anxiety and depression scale (HADS). The HADS is subjective and intended for anxiety and depression screening in patients of somatic hospitals. The scale is composed of 14 statements serving 2 subscales: subscale A–anxiety and subscale D–depression. Each statement corresponds to four variants of the answer, reflecting the gradations of the severity of the symptom and coding symptom severity from 0 points (absence) to 4 points (maximum severity). When interpreting the data, the total indicator for each subscale (A and D) is taken into account, and three ranges of values are distinguished: 0–7 points, the norm; 8–10 points, subclinical anxiety/depression; 11 points and above, clinical anxiety/depression [25].

In addition, on the day of admission, the patients subjectively assessed the severity of cough by visual analogue scales. The VAS is a ruler, which is 100 mm long, where 0 mm corresponds to the absence of cough, and 100 mm to the strongest cough.

Also, in each patient, cough was objectively assessed using a cough monitoring device developed by us [26]. The cough monitoring device is a box fastened on the patient’s body with a belt. In the device box, there are an accelerometer, a microcontroller, a breath sensor, and a microphone. The breath sensor, the microphone, and the accelerometer are connected to the microcontroller equipped with a Bluetooth module for connecting to an external computer. The breath sensor is a source of information for the change in chest circumference in the process of coughing. The accelerometer collects information about the movement of the chest during coughs. The microcontroller averages, filters, and synchronizes information from the breath sensor, the accelerometer, and audio recordings from the microphone, to identify coughs. The device works as follows: the box is placed on the patient’s body on the projection of the xiphoid process of the sternum with the help of a belt (with individual adjustment of its length for tight fixation and prevention of displacement during monitoring and patient’s movement). This placement allows the accelerometer and breath sensor to accurately track the sharp fluctuations occurring during cough, happening not only in the chest area, but also in the abdominal wall, which ensures high sensitivity regardless of the breathing type and involvement of chest or abdominal muscles. Information from these sensors and the microphone, which is fixed on the device as close as possible to the mouth, is transmitted in real time to the microcontroller, where it is averaged, filtered, and synchronized to accurately identify the cough. Using a Bluetooth wireless technology module, information can be transferred to a smartphone or a tablet computer. Information processing is carried out by a specially designed application using an amplitude filter and spectral analysis in order to eliminate noise that is not related to cough. The program also performs an automatic count of coughs [27].

A 12 h cough monitoring was carried out twice i.e, on the 1st and the 10th day of inpatient treatment. Also, on the 10th day, all patients were asked again to evaluate the severity of their cough using VAS. The severity of the cough, determined by the cough monitoring device, was expressed as the number of coughs during the monitoring period (c/d).

Statistical data processing was performed using the STATGRAPHICS 5.1 Plus for Windows software package. Quantitative data at normal distribution are presented in the form M ± σ, where M is the mean, σ is the standard deviation. Qualitative variables were compared using the χ^2^ test. To determine the correlations between the parameters, the Pearson criterion was used. Comparison of quantitative parameters was performed using one-way analysis of variance (ANOVA). Differences were considered statistically significant at a level of *p* < 0.05.

## 3. Results

At first, all the patients were divided into two groups: a group with normal body weight and a group with obesity. Normal body weight and obesity were defined according to the body mass index (BMI): 18.5–24.99 kg/m^2^ for normal body weight, 30 kg/m^2^ and more for obesity. The first group (Group 1) consisted of 53 patients with COPD and normal body weight: 34 (30.91%) men and 18 women (16.36%), mean age 60.15 ± 7.27 years. The second group (Group 2) included 57 COPD patients with obesity: 36 (32.73%) men and 22 women (20.00%), mean age 62, ± 5.24 years. All subjects had a long smoking history (the average smoking index was 15.17 ± 5.15 packs/year).

The studied groups did not differ significantly for a number of social and demographic parameters and, therefore, could be used for a comparative assessment (Table 1). The studied groups did not differ significantly by sex and age (χ^2^ = 1.253, *p* = 0.2012; F = 2.12, *p* = 0.632, respectively). Significant differences in educational level and employment status between patients of groups 1 and 2 were also not found (χ^2^ = 2.11, *p* = 0.71; χ^2^ = 1.13, *p* = 0.24, respectively). The studied groups were comparable in relation to marital status (χ^2^ = 2.23; *p* = 0.17).

According to the results of the HADS test, all patients were divided into four subgroups: 1, with normal body weight, without depression and anxiety (*n* = 30), 2, with normal body weight and subclinical/clinical depression and/or anxiety (*n* = 23), 3, with obesity without depression/anxiety (*n* = 33), 4, with obesity and subclinical/clinical depression and/or anxiety (*n* = 24). Those subgroups did not differ statistically by sex and age (χ^2^ = 2.10, *p* = 0.4241; F = 1.15, *p* = 0.1981, respectively), as well as by marital status, educational level, and employment status (χ^2^ = 1.37, *p* = 0.18; χ^2^ = 2.55, *p* = 0.17; χ^2^ = 1.11, *p* = 0.12, respectively). The HADS test results in subgroups with different body weight are presented in Table 2.

When assessing anxiety and depression, the difference in the number of points in Subgroups 1 and 2, and 3 and 4 was significant (*p* = 0.0122, *p* = 0.0167, *p* = 0.0167, *p* = 0.0310, respectively). The severity of anxiety on the HADS scale in patients with COPD and normal body weight was significantly higher than in patients with COPD and obesity and was 9.25 ± 1.37 and 8.20 ± 1.18 points, respectively (*p* = 0.0063). At the same time, the severity of depression in Subgroups 2 and 4 did not differ significantly (*p* = 0.3531).

The results obtained using VAS and the cough monitoring device are presented in Table 3. 

On the 10th day of treatment, the patients in Subgroup 1 (with normal body weight and without anxiety and/or depression) and Subgroup 3 (with obesity and without anxiety and/or depression) subjectively noted an improvement in the amount of cough compared to the 1st day of treatment. The differences in VAS values on the 1st and the 10th day were statistically significant in these subgroups (*p* = 0.0022, *p* = 0.0021, respectively). The patients in Subgroup 2 (with normal body weight and anxiety and/or depression) and Subgroup 4 (with obesity and anxiety and/or depression) reported small improvements in their cough severity; however, the mean values for VAS on day 10 did not change significantly compared to the mean values for VAS on day 1 in these subgroups (*p* = 0.1917, *p* = 0.1921, respectively). In addition, it is important to emphasize the fact that patients from Subgroup 2 had perceived their cough to be more severe on day 10 of treatment compared with the subjective assessment of cough on the 10th day in Subgroup 4. The mean value for VAS on the 10th day of treatment was 79.15 ± 7.64 mm in Subgroup 2 and 71.75 ± 6.96 mm in Subgroup 4, making these differences statistically significant (*p* = 0.0411). According to objective data, received from the cough monitor device, the number of coughs in all subgroups on day 1 did not differ significantly (*p* = 0.2012). Also, cough monitor data indicated that on the 10th day of treatment, the number of coughs has decreased in patients of all subgroups. Statistically significant differences in the number of coughs on day 10 between different subgroups were not found (*p* = 0.1813). The quantity of coughs significantly decreased on day 10 compared to day 1 of treatment in all subgroups (*p* = 0.0032, *p* = 0.0031, *p* = 0.0036, *p* = 0.0029, respectively). The association between VAS values and objective amount of cough was analyzed. In Subgroups 1 and 3, it was found that VAS values correlated positively with the actual amount of cough (*r* = 0.42, *p* = 0.0122 and *r* = 0.44, *p* = 0.0054, respectively), while in Subgroups 2 and 4, there was an inverse correlation between VAS values and coughs (*r* = −0.38, *p* = 0.0034 and *r* = −0.40, *p* = 0.0231), indicating evident differences between objective and subjective evaluation of cough in subgroups suffering from anxiety and/or depression.

## 4. Discussion

According to this study, regardless of body weight, patients with COPD and anxiety and/or depression tend to perceive the severity of their cough to be more pronounced compared to an objective evaluation. The presence of an inverse correlation between VAS values and cough monitoring device data indicates that in patients with COPD and anxiety/depression, critical thinking and assessment of their condition, in particular, of cough, is impaired. It is known that depression leads to a so-called “depressive shift” that affects the sphere of attention, thinking, memory, and prediction and it makes an assessment of a physical condition more difficult [28]. The analysis and synthesis of information is disturbed, one-sided, and incomplete, and negative and pessimistic views take over. This is why the impact of depressive disorder on the course and prognosis of somatic diseases is often discussed in different scientific fields. Recent studies examined the association between depression and widespread diseases such as diabetes, asthma, cardiovascular diseases [29,30,31]. These associations and mechanisms still remain not entirely clear, but it is evident that a combination of depression and other diseases leads to poorer health outcomes for both conditions. The fact that depression is often underdiagnosed makes it even more dangerous, because depressed patients have difficulties interacting within the society and also with health professionals, which decreases their chances of getting proper medical help. This is especially true for cases of chronic diseases, because attention is mostly paid to somatic symptoms, while the psychological state of patients is ignored, and quite often, the presence of depressive disorders remains undetermined. However, critical thinking is impaired not only in the case of depression. Anxiety disorders lead to cognitive distortions. Patients with anxiety have negative thought patterns and tend to have pessimistic views [32]. This explains the fact that in our study, patients with anxiety and/or depression could not objectively evaluate their condition, to be more precise, the severity of such a symptom as cough. According to a number of studies, depression and anxiety as comorbidities in COPD reduce adherence to pulmonary rehabilitation and medical treatment, lead to a decrease in the quality of life, increase the risk of exacerbations and mortality [33]. The importance of identifying anxiety and depressive disorders is also due to the fact that there are complex relationships between nicotine addiction, depression/anxiety disorders, and quitting smoking [34]. Prospective cohort studies show that depression and anxiety are predictors of the onset of smoking, an increase in the frequency and intensity of smoking, and a decrease in physical activity [35]. Since smoking is a risk factor for COPD, and lack of exercise is a marker of poor prognosis, these relationships are of particular importance. According to all of the above, we can state that diagnosing anxiety and/or depression is necessary.

It is also important to note that there is a pronounced connection between obesity and depression, which has been the subject of many studies. Luppino F.S. et al. conducted a meta-analysis of these studies and confirmed that obesity increases the risk of depression and depression increases the risk of obesity [24]. At the same time, the relationship between obesity and anxiety remains incompletely clear, but there is evidence that anxiety disorders such as generalized anxiety disorder and panic disorder, which significantly affect patients’ subjective assessment of their general conditions and lead to difficulties in their interactions in the society, occur in obese patients more often [36]. In turn, some studies do not confirm the presence of a significant connection between obesity and anxiety disorders [37]. In our study, the severity of anxiety in patients with normal body weight significantly exceeded the severity of anxiety in patients with obesity. This may be due to the presence of the “paradox of obesity” and the lower frequency of exacerbations in patients with COPD and obesity compared with normal-body-weight patients [20]. According to a study by Halpin D. et al., panic and fear are the predominant emotions in patients during a COPD exacerbation, even in those who are not prone to anxiety, and in some cases, they worsen the symptoms, including shortness of breath [38]. As we know, anxiety leads to twisting and distortion of information and reinforcement of negative thinking, which again leads to an increase of anxiety. Since COPD exacerbations are accompanied by fear, the frequency of these exacerbations can influence the level of anxiety, and as normal-body-weight patients have more COPD exacerbations, they would also have more severe anxiety than obese anxious patients. In addition, in our study, we observed that in patients with normal body weight and anxiety/depression, cough severity was significantly higher compared with anxious patients with obesity, who also inadequately evaluated the severity of cough when, according to the objective data, the amount decreased. This could be explained by the fact that patients with normal body weight had more severe anxiety than obese patients in this research. However, it is important to report the following limitation of our study: we combined patients with anxiety and depression and both of these conditions together, so more severe anxiety could be connected to a comorbid depression rather than to body weight and exacerbations frequency. Another shortcoming of our study is the fact that we did not include overweight subjects but only normal-body-weight and obese patients. We also did not analyze different classes of obesity. The general number of participants was not high, so further investigations are necessary.

Nevertheless, it is desirable to assess the psychological status of patients with COPD, especially in patients with normal body weight, using at least the simple and short HADS questionnaire that does not require much time to complete and interpret the results, and then refer anxious/depressive patients to a specialist. In the case of a patient suffering from anxiety and/or depressive disorder, it is preferable to use a cough monitor device as a method to objectively evaluate cough and correct the psychological status of the patient, in order to improve the prognosis and the effectiveness of therapeutic treatments.

## 5. Conclusions

Patients suffering from anxiety and/or depression in addition to COPD, regardless of body weight, cannot independently assess the severity of their condition, and therefore it is recommended to investigate the psychological status of patients with COPD and use a device to monitor cough in order to obtain factual information about the course of the disease, thus increasing the effectiveness of treatments and improving the prognosis of the disease. Special attention should be given to patients with COPD and normal body weight, since according to this study, anxious patients with normal body weight tend to exaggerate the severity of cough more than anxious patients with obesity. Further investigations in this field are highly recommended.

## Figures and Tables

**Table 1:** Comparative characteristics of patients in the groups with normal body weight (Group 1) and obesity (Group 2).

Parameter	Group 1 (*n* = 53)	Group 2 (*n* = 57)
Men, *n* (%)	34 (30.91)	36 (32.73%)
Women, *n* (%)	18 (16.36)	22 (20.00%)
Age, years (М ± σ)	60.15 ± 7.27	62.0 ± 5.24
Married, *n* (%)	42 (38.18)	47 (42.73)
Single, *n* (%)	11 (10.00)	10 (9.09)
Employed, *n* (%)	38 (34.55)	43 (39.09)
Unemployed, *n* (%)	15 (13.64)	14 (12.73)
Higher education, *n* (%)	35 (31.82)	38 (34.55)
Specialized secondary education, *n* (%)	15 (13.64)	15 (13.64)
Secondary education, *n* (%)	3 (2.73)	4 (3.64)

**Table 2:** Hospital anxiety and depression scale (HADS) test results in the studied subgroups.

Parameters	Subgroup 1 (*n* = 30)	Subgroup 2 (*n* = 23)	Subgroup 3 (*n* = 33)	Subgroup 4 (*n* = 24)
Anxiety (points)	4.95 ± 1.84	9.25 ± 1.37 ^1^	5.25 ± 1.36	8.20 ± 1.18 ^1,2^
Depression (points)	5.85 ± 2.10	10.55 ± 1.12 ^1^	6.05 ± 1.95	11.11 ± 1.88 ^1^

Footnote: data are presented as mean ± standard deviation; ^1^ the differences are significant between Subgroups 1 and 2, 3 and 4 by the level of anxiety and depression (HADS) at *p* < 0.05; ^2^ the differences are significant by the level of anxiety (HADS) between Subgroups 2 and 4 at *p* < 0.05.

**Table 3:** Comparative characteristics of objective and subjective assessment of cough severity.

Parameters	Subgroup 1 (*n* = 30)	Subgroup 2 (*n* = 23)	Subgroup 3 (*n* = 33)	Subgroup 4 (*n* = 24)
VAS, mm (1st day)	65.12 ± 10.46	86.06 ± 7.95	63.10 ± 9.86	82.12 ± 6.76
VAS, mm (10th day)	32.05 ± 11.41 ^1^	79.15 ± 7.64	36.05 ± 9.89 ^1^	71.75 ± 6.96 ^2^
Cough monitoring, c/d (1st day)	512 ± 26.44	551.08 ± 17.64	539.25 ± 16.74	511.15 ± 13.66
Cough monitoring, c/d (10th day)	80 ± 11.58 ^1^	85.33 ± 12.17 ^1^	89.15 ± 13.73 ^1^	82.75 ± 11.67 ^1^

Footnote: data are presented as mean ± standard deviation; VAS: visual analogue scale; c/d: number of coughs, ^1^ the differences are significant within groups for VAS and number of coughs at *p* < 0.05, ^2^ the differences are significant for VAS between Subgroups 2 and 4 on day 10 at *p* < 0.05.
